# Incorporation of Temperature Impact on Hot-Carrier Degradation into Compact Physics Model

**DOI:** 10.3390/mi16121424

**Published:** 2025-12-18

**Authors:** Stanislav Tyaginov, Erik Bury, Alexander Grill, Ethan Kao, An De Keersgieter, Alexander Makarov, Michiel Vandemaele, Alessio Spessot, Adrian Chasin, Ben Kaczer

**Affiliations:** 1 IMEC, Kapeldreef 75, 3001 Leuven, Belgium; erik.bury@imec.be (E.B.); alexander.grill@imec.be (A.G.); ethan.kao@imec.be (E.K.); an.dekeersgieter@imec.be (A.D.K.); alexander.makarov@imec.be (A.M.); michiel.vandemaele@imec.be (M.V.); alessio.spessot@imec.be (A.S.); adrian.chasin@imec.be (A.C.); ben.kaczer@imec.be (B.K.); 2 KU Leuven Belgium, 3001 Leuven, Belgium

**Keywords:** hot-carrier degradation, interface traps, compact physics model, Si-H bond, bond dissociation, defect generation, cryo reliability

## Abstract

We extend our compact physics model (CPM) for hot-carrier degradation (HCD) to cover the impact of ambient temperature on HCD. Three components of this impact are taken into account. First, variations in temperature perturb carrier transport. Second, the thermal component of Si-H bond rupture becomes more prominent at elevated temperatures. Third, vibrational lifetime of the bond decreases with temperature. While the first and the third mechanisms impede HCD, the second one accelerates this detrimental phenomenon. The aforementioned mechanisms are consolidated in our extended CPM, which was verified against experimental data acquired from foundry quality n-channel transistors with a gate length of 28 nm. For model validation, we use experimental data recorded using four combinations of gate and drain voltages and across a broad temperature range of 150–300 K. We demonstrate that the extended CPM is capable of reproducing measured degradation $\Delta I_{d,\mathrm{lin}}\left(t\right)$ (normalized change of the linear drain current with stress time) traces with good accuracy over a broad temperature range.

## 1. Introduction

The breath-taking scaling of the modern metal–oxide–semiconductor field-effect transistor (MOSFET)—which is the fundamental building block of the modern nano/ microelectronics—relies on careful assessment of its power, performance, and area (PPA) for design technology co-optimization (DTCO). However, hypothetically, a FET with outstanding PPA characteristics can suffer from severe degradation, thereby featuring unacceptably short device lifetime. As such, development of novel transistor architectures should include a comprehensive reliability analysis, which considers the entire complex physics of degradation. Quite unluckily, comprehensive physics-based models for FET degradation are computationally very demanding [1,2,3] and thus their application to the PPA analysis is hampered. Therefore, it is of great importance to reduce the computational burden of current technology computer-aided design (TCAD) models, while ensuring that the physical picture underlying device degradation is thoroughly incorporated, so that predictive capability of the optimized modeling approach is not jeopardized.

A few of such compact physics models (CPMs) were developed for bias temperature instability, trap-assisted tunneling, and stress-induced leakage current, which are most prominent reliability concerns in modern FETs [4,5]. Another severe degradation issue is hot-carrier degradation (HCD) [6,7], which is a complex phenomenon, because, as opposed to the aforementioned reliability concerns driven by thermalized carriers, HCD is due to non-equilibrium carriers. As a consequence, physics-based models for HCD are very complex and include—among other sub-problems—modeling of carrier transport, which is a very computationally expensive task [3,8,9,10,11,12,13,14,15,16]. Such a demand of carrier transport modeling originates from the paradigm of coupled single- and multiple-carrier (SC and MC, respectively) mechanisms of defect generation (via breakage of Si-H bonds at the $\mathrm{Si}/{\mathrm{SiO}}_{2}$ interface) induced by an ensemble of carriers shifted from equilibrium [10,11,17,18,19,20,21]. Within this paradigm, it is assumed that the SC mechanism can be initiated by a solitary hot-carrier, while the MC process is driven by colder carriers. Hence, calculation of bond breakage rates requires the carrier energy distribution function (DF). For obtaining DFs, one should solve the carrier transport problem either semiclassically [22,23] or using a quantum mechanical approach [24,25].

In order to reach a trade-off between model comprehensiveness and computational efficiency, we proposed a CPM for HCD which relies on a simplified carrier transport description [26] and which was further adapted for compact modeling at a circuit level [27]. Although our model was validated against experimental data across a broad range of gate ($V_{\mathrm{gs}}$) and drain ($V_{\mathrm{ds}}$) voltages, these data were acquired at room temperature only. To ensure the model soundness, it is important to check whether the model is valid for different temperatures. Thus, a comprehensive model should be capable of covering HCD across a broad temperature range.

The main goal of this paper is to incorporate the impact of temperature on hot-carrier degradation into our CPM. Such a CPM, which provides good accuracy (comparable to TCAD approaches) at low computational costs and is valid over a broad temperature range, will be of great importance for a fast analysis of HCD experimental data, simulations for predicting reliability of novel (not yet fabricated) FETs, and device modeling at the DTCO level. It is important to emphasize that the compact physics model developed in this work is not the same as compact models. The most crucial difference is that the CPM still relies on numerical solutions of equations, as opposed to compact models which rely on analytical formulas employed to describe device characteristics and their post-stress behavior [28,29,30]. For model validation, we employ experimental data acquired for a commercial 28 nm planar FETs with a high-*k* gate stack over a temperature (*T*) range of 150–300 K.

## 2. Experimental

**Figure 1:**
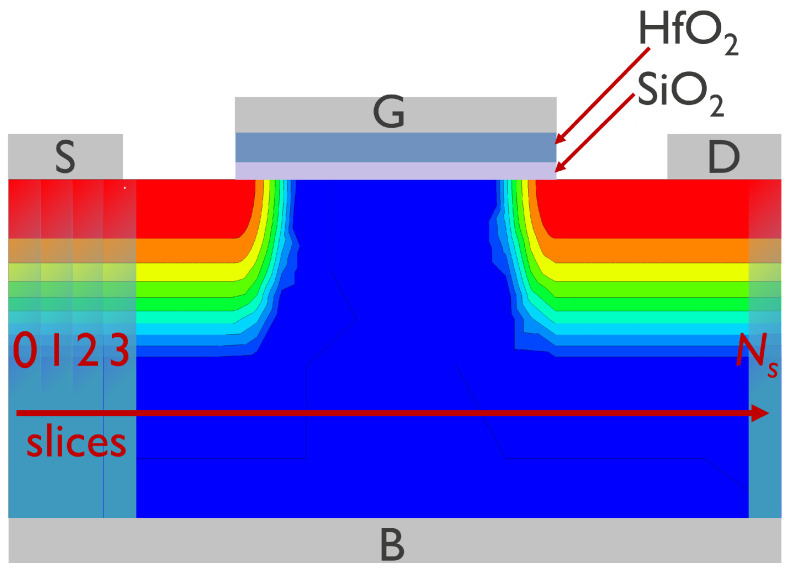
A sketch of the planar nFET used for model validation. The gate, source, drain, and bulk contacts are labeled as ‘G’, ‘S’, ‘D’, and ‘B’, respectively. Also shown are layers of silica and hafnia and a color map provides qualitative representation of the donor concentration. In our CPM, the transistor is represented as a series of slices with their enumeration beginning at the source (the slice with i=0) and ends at the drain end (the last slice has a number of $N_{s}$).

As a test vehicle for our modeling activities, we used a foundry quality planar 28 nm technology (the gate length is $L_{g}$ = 28 nm; the FET width is W=100nm). The employed transistors were n-channel FETs with an operating voltage $V_{\mathrm{dd}}$ = 1.2 V and a high-*k* gate stack containing an intermediate ${\mathrm{SiO}}_{2}$ layer followed by a ${\mathrm{HfO}}_{2}$ film; the equivalent oxide thickness is 1.3 nm. The key transistor parameters are summarized in Table 1 and a device sketch is shown in Figure 1.

These samples were subjected to HC stress under several $\{V_{\mathrm{gs}}$,$V_{\mathrm{ds}}\}$ combinations: $V_{\mathrm{gs}}=V_{\mathrm{ds}}=$ 1.8 and 2.0 V; $V_{\mathrm{gs}}=$ 1.6 V and $V_{\mathrm{ds}}=$2.0 V; $V_{\mathrm{gs}}=$ 1.49 V and $V_{\mathrm{ds}}=$2.0 V. Experiments for all pairs of stress voltages were conducted at three different temperatures, namely at 150, 225, and 300 K. As a metric of HCD, we used $\Delta I_{d,\mathrm{lin}}$, the (normalized to the time-0 value) change of the drain current in the linear regime, i.e., at the read-out voltages of $V_{\mathrm{ds}}$ = 50 mV and $V_{\mathrm{gs}}$ = 1.2 V. Maximum stress time was 143 s.

Note that studies of HCD induced time-dependent variability of device characteristics that lie outside the scope of this paper. On the other hand, such scaled FETs (as those used in our work) contain just a handful of doping atoms [31] and a comparable number of generated interface traps. These two factors—in combination with other sources of variability (fluctuations of the oxide thickness, variations in the work function, and disorder at the semiconductor/insulator interface [32,33,34,35])—result in prominent variability of time-0 characteristics as well as of degradation traces (e.g., $\Delta I_{d,\mathrm{lin}}\left(t\right)$, where *t* is stress time) [36,37,38,39,40,41,42,43]. To avoid variability, we used on-chip smart arrays [40,44], thereby enabling measurements on many samples in parallel. More precisely, for each stress condition (i.e., a combination of $V_{\mathrm{gs}}$, $V_{\mathrm{ds}}$, and *T*), we used N= 3800 samples. For every sample (labeled as *i*) and each stress time step *t*, we recorded an individual change of the drain current $\Delta I_{d,\mathrm{lin}}^{\left(i\right)}$ and then calculated the mean value as $\Delta I_{d,\mathrm{lin}}=\frac{1}{N}\sum_{i=1}^{N}\Delta I_{d,\mathrm{lin}}^{\left(i\right)}$. Throughout the paper, under $\Delta I_{d,\mathrm{lin}}$, we understand the mean value defined above.

Throughout this work, we assume that degradation is governed by dissociation of Si-H bonds at the $\mathrm{Si}/{\mathrm{SiO}}_{2}$ interface. One can envisage another degradation component, namely capture of charge carriers by oxide traps, which corresponds to bias temperature instability (BTI) [45]. BTI is known to be uniformly distributed along the interface, as opposed to HCD which is strongly localized near the drain [46,47,48]. On the other hand, post-processing of the same set of experimental data (as that used in the current study) showed strong localization of the damage for all stress conditions used in our work [40]. Indeed, the extracted factor of degradation localization was reported to lie within a range of 0.6–0.8 and these values are rather close to 1 (strong localization) than to 0 (uniform damage distribution), thereby suggesting that the BTI component can be neglected [40].

## 3. The Model

**Figure 2:**
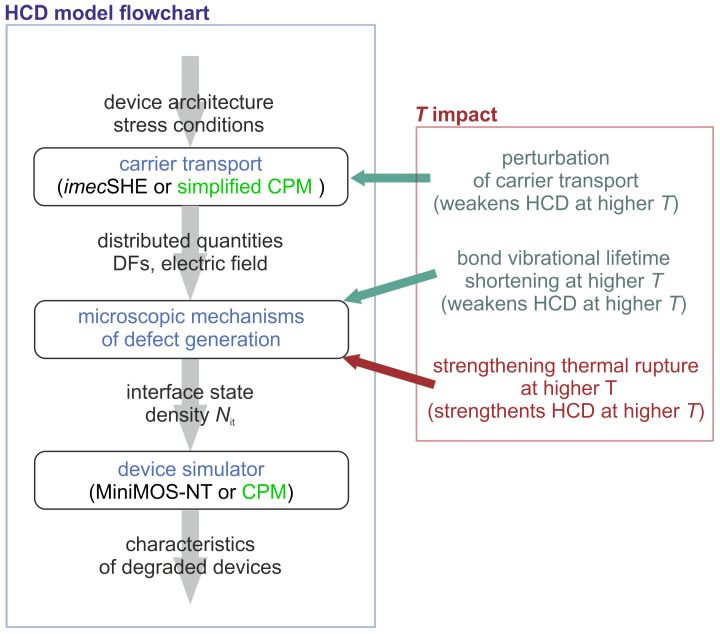
The flowchart of the HCD modeling framework. Two flavors of this framework are included in the flowchart, namely the TCAD version and the compact physics model (CPM). Both of these versions incorporate three main modules: carrier transport modeling, calculation of the defect generation rate and the interface trap density $N_{\mathrm{it}}$, and simulation of the degraded devices. The three components of temperature impact on HCD are shown, namely, perturbation of the carrier transport, shortening of vibrational lifetime of the bond at higher *T*, and acceleration of the thermal pathway of the bond dissociation reaction under elevated temperatures.

We assume that HCD is governed by dissociation of Si-H bonds at the $\mathrm{Si}/{\mathrm{SiO}}_{2}$ interface of a FET. The entire problem of HCD can be split into three main sub-problems: (i) modeling of carrier transport, (ii) calculation of bond rupture rates and the interface trap density $N_{\mathrm{it}}$, and (iii) simulation of the FET post-stress characteristics. Figure 2 depicts the flow chart of the HCD model in general case (i.e., independently, whether our TCAD model [10,11] or CPM is used). To tackle the carrier transport sub-problem, within the TCAD approach, we use the deterministic solver of the Boltzmann transport equation (BTE) *imec*SHE [49] (which is based on the open source BTE solver ViennaSHE [23,50,51]) and derive the CPM for carrier transport based on *imec*SHE results. With the carrier energy distribution functions obtained from a solution of the transport sub-problem, we proceed to calculations of defect generation rates and next to the interface trap concentration $N_{\mathrm{it}}$. This concentration is then used to simulate the characteristics of the degraded devices considering the impact of charged defects on the device electrostatics and the carrier mobility. All of these sub-problems, along with the corresponding models and methods, will be discussed in greater detail in the following subsections. The key model parameters are summarized in Table 2.

Within our modeling framework for HCD, the impact of temperature on HCD is threefold [52], see Figure 2. First, at elevated temperatures, carrier scattering is more efficient, thereby suppressing the high-energy population of the carrier ensemble and resulting in weakening of HCD [53]. Second, vibrational lifetime of the Si-H bond reduces with temperature [54]. As such, the bond returns to the ground state faster at elevated temperatures, and this factor reduces the bond dissociation rate and impedes HCD, see Section 3.2. Finally, the thermal component of bond rupture enhances at higher temperature, thereby strengthening HCD (Section 3.2).

### 3.1. Carrier Transport Modeling

In the TCAD version of our HCD model, carrier transport is tackled within the semiclassical approach, i.e., we solve the BTE for both types of carriers coupled to the Poisson equation. For this, we use the deterministic BTE solver *imec*SHE [49], which employs the spherical harmonics expansion formalism [23] and originates from the BTE solver ViennaSHE [50,51,55]. However, a direct solution of the BTE requires substantial computational resources and therefore impedes usage of TCAD models for HCD for large-scale reliability simulations at a circuit level required for predicting reliability of digital circuits.

To overcome this limitation, we developed a simplified approach to carrier transport modeling based on an analytical formula for the carrier DFs [26,56]. Namely, the DFs were represented by a simple function, which is as a superposition of the cold- and hot-carrier terms in the distribution function with three unknowns: the weights of these two terms and the effective carrier energy [57]. This approach was implemented within the ecosystem of our compact physics reliability simulator Comphy [4]. Using the Poisson solver implemented in Comphy, one can calculate the carrier concentration, the average energy of the carrier ensemble (〈E〉), and the kurtosis of the carrier distribution (based on the empirical formula by Grasser et al. [57]). However, within the first version of our CPM [56], 〈E〉 was evaluated based on the formula linking the carrier mobility (μ) and the electric field ($F_{\mathrm{ox}}$) with the average carrier energy 〈E〉. As such, 〈E〉 obtained in this fashion follows the shape of the squared electric field. At a higher abstraction level, it means that we used a simplified drift–diffusion (DD) approach to the BTE solution [58,59] to model HCD. Unfortunately, the DD model is known to be inapplicable for accurate modeling of ultra-scaled FETs [60] and HCD in these devices [61]. For instance, as we discussed in [26], the DD-based CPM for HCD results in spuriously high values of the average energy of the carrier ensemble and hence dramatically overestimates HCD.

To suppress this spuriousness, we introduced a new method for evaluating the energy 〈E〉, which is based on balancing energy gain from the applied electric field and energy loss due to various scattering mechanisms [26]. We assume that within a certain distance, the carrier ensemble gains a portion of energy determined by the electrostatic potential difference between the corresponding boundary points. As for energy loss, within every distance equal to the carrier mean free path (MFP) λ, the ensemble looses a certain portion of energy (δE). We use δE= 28 meV; detailed justification of the parameter choice is given in [26]. Regarding λ, we link it to the doping concentration $N_{\mathrm{dop}}$ via the empirical formula:

(1) $$\lambda =\lambda_{0}\left[1-\exp\left(-a/N_{\mathrm{dop}}^{1/3}\right)\right],$$

Here, $\lambda_{0}$ (= 0.05cm) is the doping-free MFP and *a* is a fitting parameter to match the value of λ = 4 nm for $N_{\mathrm{dop}}=5\times 10^{16}\;{\mathrm{cm}}^{-3}$ suggested in [62].

**Figure 3:**
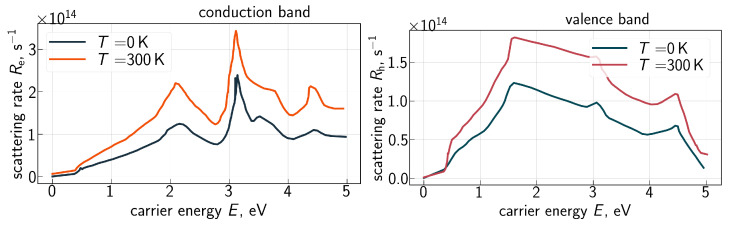
Electron–phonon scattering rates for the conduction ($R_{e}$) and valence ($R_{h}$) bands obtained from *ab initio* calculations. Data are from [63].

However, this approach does not incorporate the impact of temperature on carrier transport and therefore should be further refined. *Ab initio* calculations conducted by Qiu et al. [53] showed that the scattering rate is linearly proportional to temperature (*T*). To consider the impact of scattering on the carrier mean free path, we employ the carrier–phonon scattering rates ($R^{\left(e|h\right)}$), where index “e” labels electrons, while “h” corresponds to holes, as a function of carrier energy obtained with first principles calculations for 0 K and 300 K by Tandon et al. [63], see Figure 3. Based on these data, we calculate the mean free path for electrons/holes as

(2) $$\lambda^{\left(e|h\right)}=\frac{1}{n^{\left(e|h\right)}}\int \frac{1}{R^{\left(e|h\right)}}v^{\left(e|h\right)}\left(E\right)f^{\left(e|h\right)}\left(E\right)g^{\left(e|h\right)}\left(E\right)dE,$$

where $n^{\left(e|h\right)}$ is the carrier concentration, $v^{\left(e|h\right)}\left(E\right)$ the velocity as a function of energy *E*, $f^{\left(e|h\right)}\left(E\right)$ the occupation number, and $g^{\left(e|h\right)}\left(E\right)$ the density-of-states (DoS) in the corresponding band; the product $f^{\left(e|h\right)}\left(E\right)g^{\left(e|h\right)}\left(E\right)$ is therefore the generalized DF with the dimensionality of eV^−1^cm^−3^. The DFs are normalized as

(3) $$n^{\left(e|h\right)}=\int f^{\left(e|h\right)}\left(E\right)g^{\left(e|h\right)}\left(E\right)dE.$$

To incorporate the impact of arbitrary *T* on the mean free path of carriers, we use linear interpolation in the *T* domain of $R^{\left(e|h\right)}$ based on the spectra obtained for *T* = 0 and 300 K (Figure 3). The dependencies of the carrier velocity $v^{\left(e|h\right)}\left(E\right)$ and DoS $g^{\left(e|h\right)}\left(E\right)$ on energy are well known and were calculated by many groups; we use those from the work of Vecchi et al. [64].

**Figure 4:**
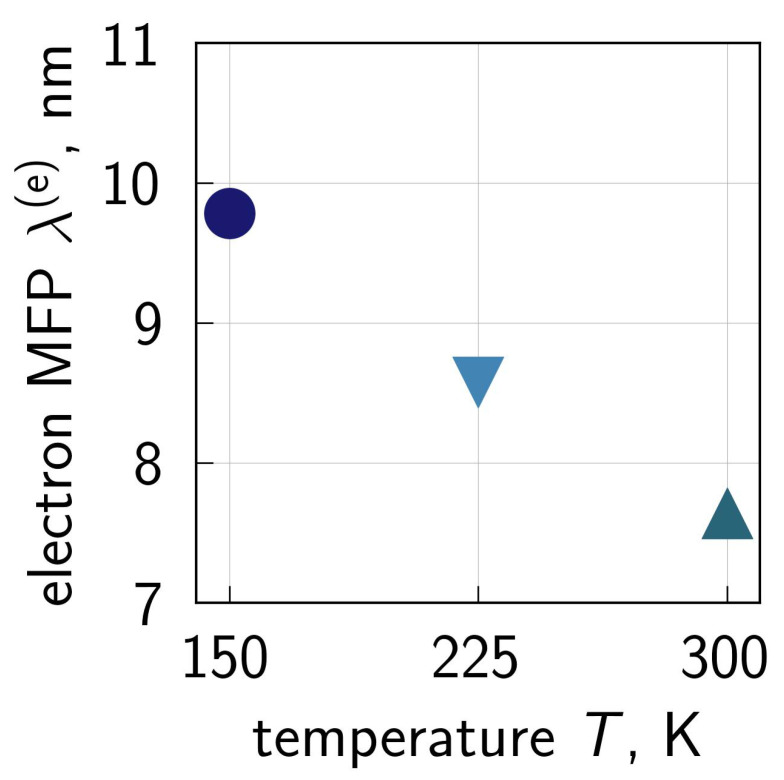
The electron mean free path calculated within our CPM for three temperatures of T= 150, 225, and 300 K and a combination of stress voltages of $V_{\mathrm{gs}}$ = $V_{\mathrm{ds}}$ = 2.0 V. Notably, different symbols and colors indicate different temperatures. The shown MFP values are obtained for the middle of the transistor, at the $\mathrm{Si}/{\mathrm{SiO}}_{2}$ interface. At this position, the component of the electric field in the transport direction is ∼$3\times 10^{5}\;$V/cm and therefore is in agreement with those extracted based on an accurate BTE solution [65].

An example of the electron MFP calculated for the three temperatures is given in Figure 4. These MFP values are shown for the device center, at the $\mathrm{Si}/{\mathrm{SiO}}_{2}$ interface, where the component of the electric field in the source–drain direction is ∼$3\times 10^{5}\;$V/cm. Our $\lambda^{\left(e\right)}$ values are consistent with those published by Fischetti and Laux in [65], where the authors—using a Monte Carlo BTE solver—obtained a dependency of the electron MFP on the electric field. One can also see that $\lambda^{\left(e\right)}$ is a decreasing function of *T* and this trend agrees with the idea that at elevated temperatures, carriers lose more energy due to different scattering mechanisms.

**Figure 5:**
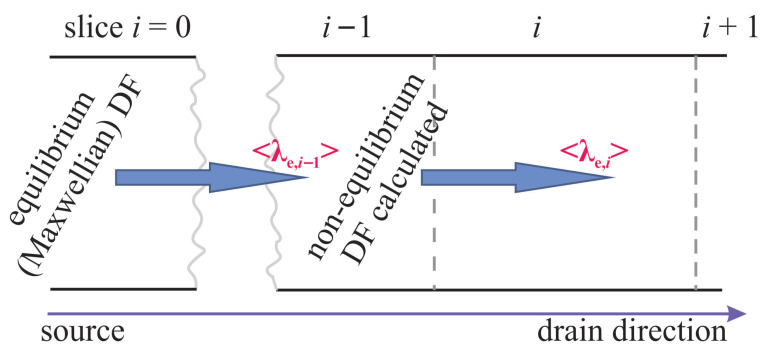
The studied FET is represented as a number of slices in the source–drain direction. We assume that in the source region, the carrier DF is Maxwellian and normalized according to (3). Therefore, based on the Maxwellian DF obtained for the slice i=0, we calculate the carrier mean free path for the next slice with i=1 using (2). With the mean free path value, we evaluate energy loss and obtain the DF for i=1. We repeat this procedure recurrently for all *i*.

However, the carrier DFs entering (2) still have to be calculated. As in our previous work focused on CPM for HCD [26,56], we represent the studied FET by a series of slices in the transport (source–drain) direction enumerated with the index *i*, Figure 5. For the slice i=0 in the beginning of the source region, we assume that the carriers are in thermal equilibrium and therefore follow a Maxwellian distribution with a normalization constant in the DF determined based on the carrier concentration, see (3). The carrier concentration is obtained from a Poisson equation solution in Comphy. Next, having the carrier DF for the slice with *i* = 0, we calculate the mean free path for the slice i=1, obtain energy loss, and calculate the non-equilibrium DF (for more detail see [26]). We repeat this procedure recurrently proceeding from a slice *i* to a slice i+1 until the DFs across the entire transistor are obtained (Figure 5).

Two important nuances are noteworthy. First, when we use (2), we do not take into account that the band structure of Si changes with *T*, i.e., we neglect the impact of *T* on the band DoS and the carrier velocity. Nevertheless, we believe that this simplification does not substantially impact our results, as many advanced transport solvers neglect these effects too [23,55]. Second, we also do not consider that phonon spectra change with *T* and—rigorously speaking—the temperature dependence of the energy loss parameter δE. However, we do take into account the temperature dependence of the carrier–phonon scattering rates and this rate already incorporates the changes of phonon parameters with temperature.

### 3.2. Modeling of Defect Generation

With the obtained carrier DFs, we proceed to calculation of bond breakage rates and the interface state density. The precursors of interface traps generated during hot-carrier stress are Si-H bonds at the $\mathrm{Si}/{\mathrm{SiO}}_{2}$ interface. These bonds were formed by anneal of the fabricated ${\mathrm{SiO}}_{2}$ film in hydrogen-reach ambient. Such a step is required to passivate Si- dangling bonds which originate from the disorder typical for the interface with amorphous dielectric [66,67,68]. These dangling bonds are amphoteric traps and therefore result in additional states in the band gap of Si, corresponding to trapped electrons or holes (with the corresponding charged defects named “P_b_-centers” [67,69,70,71,72]). In other words, these dangling bonds can accumulate charges in a FET and hence shift the device threshold voltage and reduce the carrier mobility.

**Figure 6:**
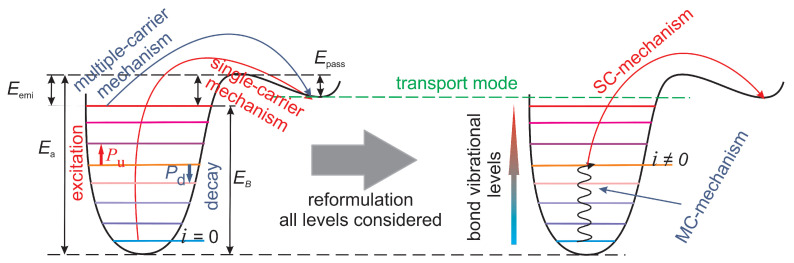
A sketch of the Si-H bond modeled within the truncated harmonic oscillator model. The left panel shows the SC and MC mechanisms of bond dissociation in a decoupled manner, while the right panel illustrates bond pre-heating by cold carriers driving the MC mechanism followed by its dissociation induced by a single highly energetical carrier, which induces an SC event.

Correspondingly, the chemical reaction underlying HCD is rupture of Si-H bonds driven by channel carriers, which results in electrically active Si- dangling bonds. There are two possible pathways of this reaction [17,18,73]. A single high-energetical electron/hole can deliver a bond-breaking portion of energy and thus dissociate a bond in a single collision, see Figure 6. This process is termed “single-carrier” mechanism of bond dissociation. However, in modern FETs with the operating voltage scaled far beyond 1.0 V, the carrier ensemble contains a negligibly small number of these carriers and thus the SC mechanism has a very low rate. Consequently, in low-voltage FETs, the bond dissociation reaction develops in the following manner: several cold carriers subsequently striking a bond can induce vibrational excitations of the bond and eventually break it (Figure 6). We refer to this mechanism as “multiple-carrier” mechanism.

Modeling of these bond dissociation mechanisms is conducted within the truncated harmonic oscillator model for the bonding potential of the Si-H bond (sketched in Figure 6), as it was suggested within the Hess model [74,75] and then extensively used by other groups [19,76]. Calculation of the rates of the SC and MC mechanisms is based on the carrier energy DF [3,8,9,10]. Namely, these rates are determined by a macroscopic quantity called “carrier acceleration integral” $\left(I_{\mathrm{SC}|\mathrm{MC}}^{\left(e|h\right)}\right)$, which, for both mechanisms, reads [77]:

(4) $$I_{\mathrm{SC}|\mathrm{MC}}^{\left(e|h\right)}=\int_{E_{\mathrm{th}}}^{\infty }f^{\left(e|h\right)}\left(E\right)g^{\left(e|h\right)}\left(E\right)\sigma_{\mathrm{SC}|\mathrm{MC}}^{\left(e|h\right)}\left(E\right)v\left(E\right)dE,$$

where $\sigma_{\mathrm{SC}|\mathrm{MC}}^{\left(e|h\right)}$ is the Keldysh-like reaction cross-section [17], which has the same mathematical expression for both SC and MC mechanisms:

(5) $$\sigma_{\mathrm{SC}|\mathrm{MC}}^{\left(e|h\right)}\left(E\right)=\sigma_{0,\mathrm{SC}|\mathrm{MC}}^{\left(e|h\right)}{\left(\frac{E-E_{\mathrm{th},\mathrm{SC}|\mathrm{MC}}}{1\;\mathrm{eV}}\right)}^{p_{\mathrm{it},\mathrm{SC}|\mathrm{MC}}}.$$

Here, $\sigma_{0,\mathrm{SC}|\mathrm{MC}}^{\left(e|h\right)}$ determines the precursor cross-section and $p_{\mathrm{it},\mathrm{SC}}$ is equal to 11, while $p_{\mathrm{it},\mathrm{MC}}=1$. Integration in (4) is carried out over the entire energy spectrum beginning at the threshold energy $E_{\mathrm{th},\mathrm{SC}|\mathrm{MC}}$, which, in case of the SC mechanism, is equal to the bonding energy $E_{a}$ and $E_{\mathrm{th},\mathrm{MC}}=\hbar \omega$, i.e., the energy distance between the oscillator vibrational levels.

The Si-H bond has two vibrational modes, namely the bending mode and the stretching mode, the energetics and properties of which were a subject of extensive research conducted by several groups [78,79,80,81,82,83]. The bending mode is characterized by $E_{a}$ = 1.5–1.6 eV, ℏω∼0.075 eV, and vibrational relaxation time of $\tau_{e}<1.0\;$ps [54,84,85]. As for the stretching mode, its parameters are $E_{a}$ = 2.5–2.8 eV [78,79,80,81,82,83], ℏω∼0.25eV, and $\tau_{e}=1.53\;$ns (at room temperature) [54]. Numerous experimental studies, however, demonstrate that the bond rupture reaction has an activation energy within a range from 2.56 [86] to 3.0 eV [87,88] and this value corresponds to the stretching mode. Also, our recent *ab initio* calculations suggest that a H release reaction developing via the bending mode does not result in energy states in the band gap of Si, thereby leaving the Si-H bond intact [68]. Therefore, in our model, it is adopted that the MC mechanism occurs via the stretching mode with $E_{a}$ = 2.75 eV. Let us finally note that, due to the amorphous nature of ${\mathrm{SiO}}_{2}$ and hence structural disorder at the $\mathrm{Si}/{\mathrm{SiO}}_{2}$ interface [67], the bonding energy is a normally distributed quantity and (in addition to the mean value $E_{a}$) is characterized by a standard deviation $\sigma_{E}$ [68,88]. For these calculations, we use $\sigma_{E}$ = 0.52 eV, which is a higher value compared to that obtained with *ab initio* calculations in our previous work [68].

We consider all possible superpositions of the SC and MC mechanisms [10,11,20]. That is, a Si-H bond can be first excited by several cold carriers to an excited state *i* with the energy $E_{i}$ (bond excitation via the MC process) and then dissociated by a single highly energetical carrier (SC process), which delivers a bond-breaking portion of energy to the bond (this energy is substantially lower than $E_{a}$ required to dissociate the bond from the ground state). If the bond is first excited by the MC mechanism, the barrier between the bonded state *i* and the transport mode is reduced by $E_{i}$, and the acceleration integral for the SC process occurring from the level *i* is

(6) $$I_{\mathrm{SC},i}=\int f\left(E\right)g\left(E\right)\sigma_{0}{\left(E-E_{a}+E_{i}\right)}^{p_{\mathrm{it},\mathrm{SC}}}v\left(E\right)dE.$$

This formula is valid for both electrons and holes and therefore, from here on, we omit indexes “e” and “h”.

For each level *i*, the corresponding contribution to the entire bond rupture rate is calculated as

(7) $$R_{\mathrm{SC},i}=w_{\mathrm{th}}\exp\left[-\left(E_{a}-E_{i}\right)/k_{B}T_{L}\right]+I_{\mathrm{SC},i},$$

where the first term (with the corresponding attempt frequency $w_{\mathrm{th}}$) represents the thermal activation of H over the corresponding potential barrier between the level *i* and the transport mode, while the acceleration integral $I_{\mathrm{SC},i}$ corresponds to the HC contribution to bond dissociation.

Let us emphasize that the first term in (7) is a rapid function of energy and therefore heating the transistor results in substantial strengthening of the thermally activated bond breakage. Such acceleration of degradation is especially prominent when the cold carrier flux is large (high value of $I_{\mathrm{SC}}$) and therefore the highest vibrational levels of the bond have large occupation numbers. This mechanism enhances HCD at elevated *T*, as opposed to accelerated scattering resulting in HCD suppression, see Section 3.1.

The kinetics of the bond breakage reaction is described by a system of first order rate equations written for each bonded state:

(8) $$\begin{array}{c} \frac{dn_{0}}{dt}=P_{d}n_{1}-P_{u}n_{0}-R_{a,0}n_{0}+R_{p,0}N_{\mathrm{it}}^{2}\\ \frac{dn_{i}}{dt}=P_{d}\left(n_{i+1}-n_{i}\right)-P_{u}\left(n_{i}-n_{i-1}\right)-R_{a,i}n_{i}+R_{p,i}N_{\mathrm{it}}^{2}\\ \frac{dn_{N_{l}}}{dt}=P_{u}n_{N_{l-1}}-P_{d}n_{N_{l}}-R_{a,N_{l}}n_{N_{l}}+R_{p,N_{l}}N_{\mathrm{it}}^{2}. \end{array}$$

In (8), $n_{i}$ is the vibrational level occupancy and $N_{l}$ is the index of the last bonded level. The bond excitation/relaxation rates $P_{u}$ and $P_{d}$ read as

(9) $$\begin{array}{c} P_{u}=\omega_{e}\exp\left(-\hbar \omega /k_{B}T_{L}\right)+I_{\mathrm{MC}},\\ P_{d}=\omega_{e}+I_{\mathrm{MC}}. \end{array}$$

Here, $\omega_{e}=1/\tau_{e}$ with $\tau_{e}$ being vibrational lifetime of the bond; the term $I_{\mathrm{MC}}$ corresponds to the bond excitation induced by the channel carriers.

As it was demonstrated by Andrianov et al. with quantum chemistry calculations [54], lifetime $\tau_{e}$ is a decreasing function of *T*. For instance, at room temperature, $\tau_{e}$ is ∼1.5 ns, while at 125 °C, this lifetimes shrinks to ∼1.25 ns. To incorporate the impact of temperature on bond vibrational lifetime, we use the $\tau_{e}\left(T\right)$ dependency calculated in [54] across a broad temperature range. Such a lifetime behavior leads to weakening of HCD at higher *T*, because the Si-H bond faster returns to equilibrium.

We solve system (8), assuming the time-scale hierarchy, i.e., considering that typically vibrational lifetime $\tau_{e}$ is much shorter than the corresponding times for bond breakage. Within this approach, system (8) simplifies to a single differential equation:

(10) $$\frac{dN_{\mathrm{it}}}{dt}=\left(N_{0}-N_{\mathrm{it}}\right)R_{a}-N_{\mathrm{it}}^{2}R_{p},$$

where $N_{0}$ is the concentration of intact Si-H bonds and $R_{a}$ is the cumulative bond rupture rate

(11) $$\begin{array}{c} R_{a}=\frac{1}{k}\sum_{i}R_{a,i}{\left(\frac{P_{u}}{P_{d}}\right)}^{i}, \end{array}$$

evaluated by summation over all bond levels considering their occupation probabilities

(12) $$n_{i}=k{\left(\frac{P_{u}}{P_{d}}\right)}^{i},$$

with *k* being a normalization coefficient to ensure $\sum n_{i}=N_{0}$ and is written as

(13) $$k=N_{0}\sum_{i}{\left(\frac{P_{u}}{P_{d}}\right)}^{i}.$$

As for the passivation reaction, its rate $R_{p}$ corresponds to thermal activation over a potential barrier $E_{\mathrm{pass}}$ and is determined by the Arrhenius law:

(14) $$R_{p}=\nu_{p}\exp\left(-E_{\mathrm{pass}}/k_{B}T_{L}\right)/N_{0},$$

where $\nu_{p}$ is the attempt frequency; $N_{0}$ in the denominator is needed for dimensionality consistency in (10). The value of $E_{\mathrm{pass}}$ used in our model is 1.75 eV and is consistent with experimental values extracted by different groups [89,90,91,92,93]. However, in the context of this paper and given that the temperature range is limited by 300 K, the passivation reaction rate can be neglected (however, it was implemented in our CPM). This is because, as it was shown previously [93], this reaction has a significant rate at temperatures of ∼420 K and higher.

Finally, the system (8) simplified to (10) has an analytical solution:

(15) $$\begin{array}{c} N_{\mathrm{it}}\left(t\right)=\frac{\sqrt{R_{a}^{2}/4+N_{0}R_{a}R_{p}}}{R_{p}}\;\frac{1-\tilde{f}\left(t\right)}{1+\tilde{f}\left(t\right)}-\frac{R_{a}}{2R_{p}},\\ \tilde{f}\left(t\right)=\frac{\sqrt{R_{a}^{2}/4+N_{0}R_{a}R_{p}}-R_{a}/2}{\sqrt{R_{a}^{2}/4+N_{0}R_{a}R_{p}}+R_{a}/2}\;\;\exp\left(-2t\sqrt{R_{a}^{2}/4+N_{0}R_{a}R_{p}}\right). \end{array}$$

In this formulation, the time dependency of $N_{\mathrm{it}}\left(t\right)$ is determined by $\tilde{f}\left(t\right)$.

### 3.3. Modeling of Degraded FETs

With the obtained $N_{\mathrm{it}}\left(t\right)$, we can simulate characteristics of the degraded devices for arbitrary stress time. Note that the impact of the charged interface traps on FET performance is twofold. First, these defects distort the band diagram of the transistor and lead to a threshold voltage shift. Second, they scatter carriers in the channel and therefore reduce the carrier mobility. Both contributions to HCD are taken into account in our CPM for HCD [26,94].

As the HCD metric is the change of the drain current (ΔI), we calculate this quantity as

(16) $$\begin{array}{c} \begin{array}{c} I=\sigma V_{\mathrm{ds}}\\ \Delta I=\Delta \left(\sigma \right)V_{\mathrm{ds}}. \end{array} \end{array}$$

Here, σ denotes the total FET conductivity. As mentioned before, the transistor is represented by a series of slices and each slice *i* has a conductivity $\sigma_{i}$. Given that, the FET conductivity reads

(17) $$\begin{array}{c} \sigma ={\left[\sum_{i}1/\sigma_{i}\right]}^{-1}={\left[\sum_{i}\frac{1}{qn_{i}\mu_{i}}\right]}^{-1}. \end{array}$$

The electron concentration in the slice *i* is denoted as $n_{i}$ and $\mu_{i}$ is the carrier mobility of this slice; *q* is the absolute value of the electron charge. By combining (16) and (17), we obtain the drain current change

(18) $$\begin{array}{c} \Delta I∼\Delta \left[{\left(\sum_{i}\frac{1}{qn_{i}\mu_{i}}\right)}^{-1}\right]V_{\mathrm{ds}} \end{array}$$

The perturbation of the electrostatic potential due to created $N_{\mathrm{it}}$ is obtained within a simplified solution of the Poisson equation using the Pregaldiny model [95,96]. Namely, in each slice, the effective gate voltage drift is calculated

(19) $$\begin{array}{c} \delta V_{\mathrm{gs},i}∼N_{\mathrm{it},i}/C_{\mathrm{ox}}, \end{array}$$

where $N_{\mathrm{it},i}$ is the interface trap density in the slice *i* and $C_{\mathrm{ox}}$ is the sheet capacitance. Based on the corrected $V_{\mathrm{gs}}$ value, we calculate other relevant quantities such as the band bending profile, carrier concentration, etc.

To cover the second component of the charged trap impact on device performance, we calculate the degraded mobility ($\mu_{\mathrm{degr}}$) using the empirical formula, as suggested in [97,98]:

(20) $$\begin{array}{c} \mu_{\mathrm{degr}}=\frac{\mu_{\mathrm{fresh}}}{\left(1+\alpha N_{\mathrm{it}}\right)}. \end{array}$$

Here, the parameter α = 10^−13^ cm^−2^ determines the magnitude of the impact of $N_{\mathrm{it}}$ on $\mu_{\mathrm{degr}}$ and $\mu_{\mathrm{fresh}}$ is the mobility in the intact transistor. For calculating $\mu_{\mathrm{fresh}}$, we employ the standard low- and high-field mobility models [99,100], which are commonly used in commercial device simulators such as, e.g., MINIMOS-NT in the GlobalTCADSolutions environment [101].

It is noteworthy that the TCAD version of the model (used to derive the current CPM) was a subject of careful verification using different transistor architectures and stress conditions. In addition to reproducing convenient metrics of HCD (such as changes of the drain current in the linear and saturation regimes) [10,11], we performed a comparison of $N_{\mathrm{it}}\left(x\right)$ profiles calculated within our model against those obtained with charge-pumping measurements [102]. In this study, good agreement between simulated and measured $N_{\mathrm{it}}\left(x\right)$ dependencies was demonstrated, thereby proving soundness of our model also at the microscopic level, in addition to the device level.

### 3.4. Model Limitations

Model limitations can be conditionally split into two categories: (1) limitations related to the TCAD version of our HCD model and (2) limitations related to the simplifications applied within the CPM. Let us begin with the first category.

The TCAD version of our HCD model employs a semiclassical approach to the transport problem, which has limited applicability at low temperatures and has been lacking some important transport peculiarities. Among them, a very crucial phenomenon is that at cryo *T*, a significant contribution to carrier transport is given by conduction/valence DoS tails propagating inside the bandgap of Si. This is tightly related to the subthreshold swing saturation, which usually manifests itself at critical *T* between 50 and 100 K. However, band-tail transport in Si should essentially only play a significant role for small to moderate gate voltages, that is not for high-inversion typical for the HCD conditions we focus on. More details can be found in [103], as this work presents a compact model for the band-tail contribution and can be adopted for an extended (below 50 K) version of our CPM. Furthermore, Oka et al. [104] reported evidence that band-tails should only play a significant role below a few tens of K. Quite the same conclusion was reported by Wang et al. [105].

The next model limitation stems from self-heating [106,107], which is neglected in our CPM and whose impact on HCD becomes especially important at cryo temperatures. Among these lines, recently, Cassé et al. demonstrated substantial temperature increase (induced by self-heating) of more than 50 K at ambient temperature of 4 K in STMicroelectronics 28 nm fully depleted silicon-on-insulator transistors even at moderate power dissipated within the device [108]. These results are strong evidence that at cryo temperatures self-heating should be taken into account.

In this study, self-heating is neglected due to the following reason. Recent multi-scale thermal simulations performed for a high performance CPU with corresponding workloads [109] showed that the characteristic distance of temperature spatial variations (due to self-heating) is of an order of a few micrometers. As such, a single transistor does not have a significant temperature gradient even though various parts of the circuit can be at substantially different temperatures. Instead, a FET can be assigned effective temperature, which does not vary within its channel, but can depend on the location on the wafer. However, such a scenario is properly captured by our CPM.

In addition to these two limitations, the TCAD model uses the Maxwell–Boltzmann (MB) statistics, which is also a simplification, as a more accurate solution of the transport sub-problem can be obtained with the Fermi–Dirac (FD) statistics. An MB-based approach tends to overestimate the low-energy part of the DF and slightly underestimate its values within the high-energy tail. The DFs inside the channel are much less affected. However, because the MFP is inversely proportional to the scattering rate, and the dominant phonon scattering rate in the channel increases with carrier energy, low-energy carriers contribute disproportionately to the effective MFP. If low-energy carriers are overpopulated (as with MB DFs), the effective MFP will be overestimated. Therefore, one may envisage that the MB statistics should systematically overestimate the MFP in deterministic BTE simulations.

This overestimation of MFP (due to the overestimation of low-energy carrier population) is present at all temperatures, so the qualitative trend is largely preserved. At lower temperatures, the overestimation of MFP should become more pronounced, but we still expect that the physical behavior remains the same, i.e., as temperature decreases, the carrier population shifts to lower energies, the MFP increases, and transport in the channel becomes more ballistic. In our HCD framework, a larger MFP means that carriers lose energy more slowly in the channel, so their kinetic energy (and thus HCD) is higher. An MB-based model will exaggerate this by overestimating MFP, and therefore may insignificantly overestimate HCD, but the temperature trend of HCD should hold. An approach to reconciliation of this issue, i.e., towards adaptation of BTE solvers for using the FD statistics was recently presented by Renner et al. [110].

Regarding drawbacks arising from the CPM itself, the main simplification is related to linear interpolation of the scattering rate over the temperature domain. The idea that this rate is linearly proportional to *T* was expressed in [53] and this publication shows that the $R^{\left(e|h\right)}\left(T\right)$ dependency becomes substantially non-linear at temperatures of ∼150 K and below. However, the structure of our CPM allows one to load scattering rates computed at arbitrary temperature (using e.g., first principles calculations) and therefore overcome this model drawback.

To summarize, the CPM presented here is valid at temperatures above 50 K. As for the upper limit, we do not envisage any limitations within the range of typical operating conditions of microelectronic components. One should emphasize that at ∼420 K and above, the reaction of passivation of Si- dangling bonds has a significant rate, but this process is captured by our model.

## 4. Results and Discussion

**Figure 7:**
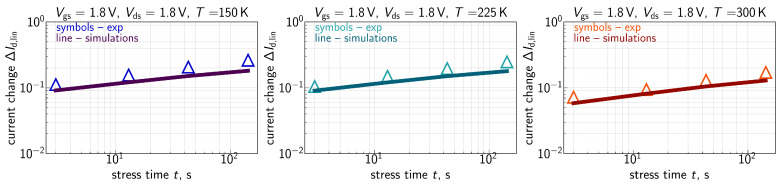
Experimental (symbols) and simulated (lines) with the CPM for HCD $\Delta I_{d,\mathrm{lin}}\left(t\right)$ traces ($\Delta I_{d,\mathrm{lin}}$ are relative, i.e., normalized to the drain current of the pristine FET) for three different temperatures: 150 K (**left panel**), 225 K (**central panel**), and 300 K (**right panel**). The stress voltages are $V_{\mathrm{gs}}$ = $V_{\mathrm{ds}}$ = 1.8 V. One can see that the model accurately reproduces experimental $\Delta I_{d,\mathrm{lin}}\left(t\right)$ curves.

**Figure 8:**
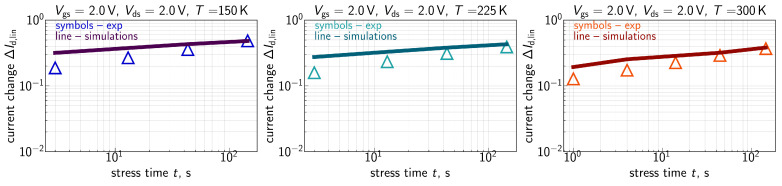
Same as in Figure 7, but for $V_{\mathrm{gs}}$ = $V_{\mathrm{ds}}$ = 2.0 V.

**Figure 9:**
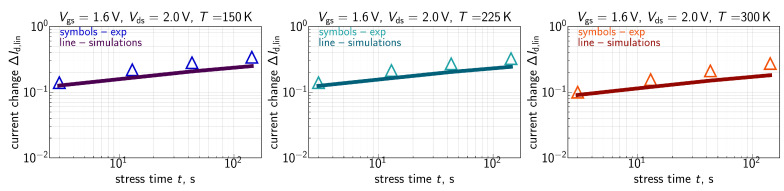
Same as in Figure 7, but for $V_{\mathrm{gs}}$ = 1.6 V and $V_{\mathrm{ds}}$ = 2.0 V.

**Figure 10:**
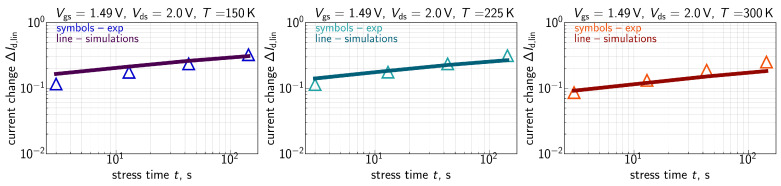
Same as in Figure 7, but for $V_{\mathrm{gs}}$ = 1.49 V and $V_{\mathrm{ds}}$ = 2.0 V.

The measured $\Delta I_{d,\mathrm{lin}}\left(t\right)$ traces for all the three environment temperatures are shown in Figure 7 for $V_{\mathrm{gs}}=V_{\mathrm{ds}}=$ 1.8 V, Figure 8 for $V_{\mathrm{gs}}=V_{\mathrm{ds}}=$ 2.0 V, Figure 9 for $V_{\mathrm{gs}}=$ 1.6 V and $V_{\mathrm{ds}}=$2.0 V, and Figure 10 for $V_{\mathrm{gs}}=$ 1.49 V and $V_{\mathrm{ds}}=$2.0 V. One can see that for these combinations of $\{V_{\mathrm{gs}}$,$V_{\mathrm{ds}}\}$, $\Delta I_{d,\mathrm{lin}}$ values become larger at lower temperatures.

At a first glance, this behavior contradicts the commonly acknowledged concept that HCD is accelerated at elevated temperatures in short-channel FETs, while in longer-channel counterparts, HCD is mitigated at higher *T* [19,75,111,112,113,114]. Alleviation of HCD in longer-channel FETs is attributed to more efficient scattering under increased temperature, resulting in depopulation of high-energy fraction of the carrier ensemble. This behavior was reported by several groups, which performed a numerical solution of the BTE by means of a Monte Carlo approach [115,116,117]. As for HCD enhancement at higher *T*, the reason is still vague. Some groups [21] suggest that this behavior is determined by electron–electron scattering which catalyzes HCD. Other groups [114] link the enhancement of HCD at increased *T* with trapping of charge carriers by pre-existing oxide traps (this mechanism is of the same microscopic origin as bias temperature instability); this process is known to have a strong temperature dependency [118]. The transition between the two aforementioned trends occurs within a gate length range of 100–180 nm and therefore in our nFETs, one may expect that $\Delta I_{d,\mathrm{lin}}\left(T\right)$ is an increasing function. However, relatively recently, several groups reported [11,40,119] that the temperature dependency of HCD can be non-monotonic and even the same FET can feature HCD suppression and activation at higher temperatures, depending on the applied $\{V_{\mathrm{gs}}$,$V_{\mathrm{ds}}\}$. Therefore, we conclude that the obtained $\Delta I_{d,\mathrm{lin}}\left(t\right)$ behavior is reasonable.

Another important peculiarity of the measured traces is a smaller—compared to values shown by other groups [76,120]—time exponent, which is 0.2–0.35. Previously, we reported and modeled flat $\Delta I_{d,\mathrm{lin}}\left(t\right)$ traces [11,121] and a smaller slope was explained to be due to very high (compared to $V_{\mathrm{dd}}$) stress voltages $V_{\mathrm{gs}}$ and $V_{\mathrm{ds}}$. Under these aggressive stress conditions, all Si-H bonds in the drain region are predominantly broken already at short stress times, i.e., the concentration $N_{\mathrm{it}}$ is saturated. Therefore, further development of HCD is due to propagation of the $N_{\mathrm{it}}$ front inside the channel and this process has a smaller time exponent. A very similar trend was reported by Varghese et al. [122]: the time exponent of HCD induced degradation traces decreases at higher voltages and/or long stress times. These severe stress conditions used in this work were chosen intentionally to ensure that both SC and MC mechanisms of bond rupture are prominent and we can benchmark the model in an intricate regime when all HCD components have high rates.

**Figure 11:**
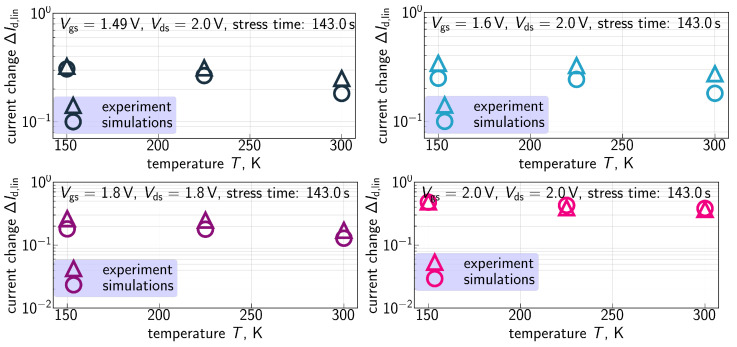
Experimental and calculated $\Delta I_{d,\mathrm{lin}}\left(T\right)$ dependencies obtained for all the combinations of stress voltages and the stress time step of 143s. One can see that the model can reproduce $\Delta I_{d,\mathrm{lin}}$ values with good agreement for all shown cases. Noteworthy is that $\Delta I_{d,\mathrm{lin}}\left(T\right)$ is a decreasing function of *T* and the extended CPM captures this behavior.

$\Delta I_{d,\mathrm{lin}}\left(t\right)$ traces calculated with the extended compact physics model for HCD are also shown in Figure 7, Figure 8, Figure 9 and Figure 10 for all considered temperatures. One can see that good agreement between experimental and simulated $\Delta I_{d,\mathrm{lin}}\left(t\right)$ curves across the entire temperature range was achieved. To better resolve the temperature dependency of $\Delta I_{d,\mathrm{lin}}$, we plot them as a function of *T* in Figure 11 for all combinations of $\left\{V_{\mathrm{gs}},V_{\mathrm{ds}}\right\}$ and a fixed stress time step of 143s. Consistently, with data shown in Figure 7, Figure 8, Figure 9 and Figure 10, $\Delta I_{d,\mathrm{lin}}$ values plotted in Figure 11 reduce with increasing temperature.

This trend can be explained, keeping in mind the three aforementioned mechanisms of the *T* impact on HCD—perturbation of carrier transport, enhancement of the thermal component of bond dissociation at higher *T*, and reduction in bond vibrational lifetime. Therefore, the experimentally obtained behavior of $\Delta I_{d,\mathrm{lin}}\left(T\right)$ suggests that in these FETs, the temperature dependence of HCD is dominated by carrier transport combined with with the shortening of vibrational lifetime rather than the thermal component of bond breakage. This is because, as we already mentioned, higher scattering rates at elevated temperatures result in depopulation of the high energy population of the carrier ensemble and therefore suppression of HCD. If vibrational lifetime of the bond becomes shorter, its vibrational modes decay faster, thereby reducing the MC process rate and weakening HCD. To the contrary, the thermal process of interface trap creation has a higher rate at increased *T* and therefore strengthens HCD. In our previous papers [52,94], we discussed that this mechanism has a smaller (compared to the two other components) impact on HCD. To summarize, in the studied FETs subjected to HCD at the given stress conditions, HCD weakening due to transport perturbation prevails over HCD acceleration via the thermal component of the bond rupture reaction.

**Figure 12:**
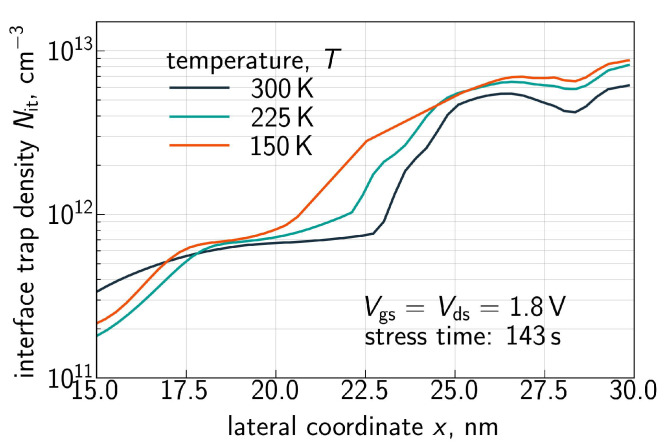
The interface trap density $N_{\mathrm{it}}$ as a function of the coordinate *x* along the $\mathrm{Si}/{\mathrm{SiO}}_{2}$ interface in the source–drain direction (drain is at the right side of the graph) plotted for $V_{\mathrm{gs}}$ = $V_{\mathrm{ds}}$ = 1.8 V, three different temperatures of 150, 225, and 300 K, and the stress time step of 143 s. Decreasing the *T* results in a larger $N_{\mathrm{it}}$ peak at the drain.

Finally, Figure 12 shows three interface trap density $N_{\mathrm{it}}\left(x\right)$ profiles (*x* is the coordinate in the source–drain direction, and *x* = 28 nm corresponds to the FET drain) computed with the CPM for $V_{\mathrm{gs}}$ = $V_{\mathrm{ds}}$ = 1.8V and the three temperatures (150, 225, and 300 K). In order to better resolve the temperature behavior of $N_{\mathrm{it}}$, the profiles are shown for the drain-related half of the FET. First of all, $N_{\mathrm{it}}$ features a peak near the drain end of the gate electrode and this behavior is consistent with one of the main peculiarities of HCD—its strong localization at the drain side of the device [46,47,48]. Second, the near-drain $N_{\mathrm{it}}$ peak becomes more prominent at lower temperatures and this behavior is consistent with the data set depicted in Figure 7, Figure 8, Figure 9 and Figure 10.

As discussed above, one can see that the $N_{\mathrm{it}}\left(x\right)$ profile is flat in the drain region of the FET. This behavior is visible for all three temperatures. At lower *T*, the $N_{\mathrm{it}}$ profile smears out over the channel in a more prominent manner, thereby leading to larger $\Delta I_{d,\mathrm{lin}}$ values. The same is applicable to the time dependency of HCD—after saturation of $N_{\mathrm{it}}$ in the drain area, further increase in $\Delta I_{d,\mathrm{lin}}$ with *t* is determined by $N_{\mathrm{it}}$ propagation towards the source; this behavior explains the small time exponents of 0.2–0.35 seen in Figure 7, Figure 8, Figure 9 and Figure 10.

**Figure 13:**
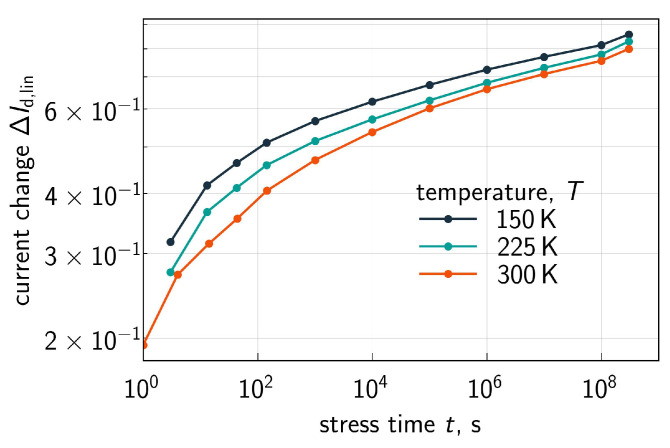
$\Delta I_{d,\mathrm{lin}}\left(t\right)$ dependencies evaluated for over a broad stress time window: $t\in [1.0;\;\;\;3\times 10^{8}]\;$s. Stress voltages are $V_{\mathrm{gs}}$ = $V_{\mathrm{ds}}$ = 2.0 V. Data are shown for all three temperatures.

To demonstrate predictive capabilities of the developed CPM, we calculate $\Delta I_{d,\mathrm{lin}}\left(t\right)$ dependencies for $V_{\mathrm{gs}}$ = $V_{\mathrm{ds}}$ = 2.0V and all three temperatures for stress times of up to 10years, Figure 13. It is noteworthy that, due to a relatively small time slope, $\Delta I_{d,\mathrm{lin}}$ does not dramatically change even within several decades of time variation. Also, the temperature trend holds true at 10 years.

Finally, we would like to emphasize that the CPM presented in this work cannot be treated as a compact model. Quite to the contrary to compact models, the CPM does not focus on linking the time dependence of degradation parameters (e.g., the threshold voltage shift and the drain current change) with the operating conditions, primarily $V_{\mathrm{gs}}$, $V_{\mathrm{ds}}$, and *T*, using a close-form analytical expression. Instead, the CPM still relies on numerical solutions of such equations as the Poisson equation and a system of equations required to obtain the carrier DFs, etc. However, these solutions do not demand substantial computational resources and therefore, we believe that our approach provides a good trade-off between modeling accuracy (which is comparable to TCAD models) and computational burden. A niche of applicability for the CPM is supposed to be related to optimization of device architecture at the DTCO level focused on alleviation of HCD, as well as providing simulation support (instead of much more expensive TCAD models) for deriving a reliability compact model in a manner similar to that presented in [27].

Another possible area of CPM applicability is consolidation within the compact modeling framework for transistor aging published in our previous work [123]. This framework uses a simplified version of the Enz–Krummenacher–Vittoz (EKV) model [124,125] to evaluate current–voltages characteristics of pristine FETs and transistors subjected to HCD and BTI; in [123], its careful validation was carried out and good agreement between experimental and calculated transistor degradation characteristics was shown. Notably, the HCD simulation module of this framework employs a very similar (to that used in the current CPM) approach to a simplified description of carrier DFs using an analytical formula. However, our EKV-based approach has two substantial limitations. First, it still relies on data obtained with TCAD simulations, required to calibrate this framework. Second, HCD and BTI modeling was not carried out for such low temperatures as 150 K. We believe that the CPM presented in this work should allow us to reconcile these limitations.

## 5. Conclusions

We have developed and validated a compact physics model for hot-carrier degradation, which takes into account the impact of temperature on this detrimental phenomenon. To validate this model, we used foundry quality n-channel planar FETs with a gate length of 28 nm. The impact of varying temperature on HCD is threefold: (i) variations in temperature disturb carrier transport (thereby weakening HCD), (ii) it shortens vibrational lifetime of the bond (this mechanism also impedes HCD as the bond faster returns to its equilibrium), and (iii) higher *T* enhances the thermal component of bond dissociation (this mechanism accelerates HCD). All these mechanisms were implemented in the refined CPM for HCD.

The major extension of the CPM is related to incorporation of strengthened (at higher *T*) carrier–phonon scattering within simplified carrier transport treatment. For achieving this goal, for each slice of the transistor (in the CPM for HCD the transistor is represented as number of slices in the source–drain direction) we calculate the carrier mean free path based on the carrier energy distribution function obtained for the previous slice. The procedure is repeated recurrently, starting at the first slice corresponding to the source region of the FET, where the carriers are thermalized and thus follow a Maxwellian distribution. Calculation of the mean free path is based on the look-up table for the scattering rate, which was taken from an *ab initio* paper [63].

Finally, our model was shown to accurately reproduce experimental $\Delta I_{d,\mathrm{lin}}\left(t\right)$ traces and capture the temperature behavior of HCD. Our CPM was discussed to be valid across a temperature range from ∼50 K and up to the highest temperatures typical for MOSFET operating conditions.

## Figures and Tables

**Table 1:** Main architectural parameters of the planar n-channel FET used in this work.

Parameter	Value	Description
$L_{g}$	28 nm	gate length
*W*	100 nm	transistor width
EOT	1.3 nm	equivalent oxide thickness
$V_{\mathrm{dd}}$	1.2 V	operating voltage

**Table 2:** Main parameters of the presented model split into three categories: carrier transport, defect generation, and degraded device modeling.

Parameter	Value	Description
δE	28 meV	carrier energy loss
$\lambda_{0}$	0.05 cm	doping-free mean free path
$E_{a}$	2.75 eV	Si-H bonding energy (mean value)
$\sigma_{a}$	0.52 eV	standard deviation of bonding energy
ℏω	0.25 eV	energetic distance between vibrational levels of Si-H
$E_{\mathrm{pass}}$	1.75 eV	energy barrier for the passivation reaction
$\sigma_{0,SC}$	1.5 × 10^−19^ cm^2^	cross-section of the SC process
$\sigma_{0,MC}$	1.5 × 10^−20^ cm^2^	cross-section of the MC process
$N_{0}$	1.2 × 10^13^ cm^−3^	density of intact Si-H bonds
α	10^−13^ cm^−2^	$N_{\mathrm{it}}$ induced mobility degradation magnitude

## Data Availability

The data presented in this study are available on request from the corresponding author.

## Abbreviations

The following abbreviations are used in this manuscript:

BTE	Boltzmann transport equation
BTI	Bias temperature instability
CPM	Compact Physics Model
DD	Drift–diffusion (appear to carrier transport modeling)
DF	Distribution function
DoS	Density-of-states
DTCO	Design technology co-optimization
EKV	Enz–Krummenacher–Vittoz (model)
FD	Fermi–Dirac (statistics)
HCD	Hot-Carrier Degradation
MB	Maxwell–Boltzmann (statistics)
MC	Multiple-carrier (mechanism of bond dissociation)
MFP	mean free path (of carriers)
MOSFET	Metal–Oxide–Semiconductor Field-Effect Transistor
PPA	Power, performance, area
SC	Single-carrier (mechanism of bond dissociation)
TCAD	Technology computer-aided design
WCC	Worst-Case Conditions (of hot-carrier degradation)
